# Immunohistochemical Evaluation of Cell Cycle Regulators: Impact on Predicting Prognosis in Stage T1 Urinary Bladder Cancer

**DOI:** 10.5402/2012/379081

**Published:** 2012-12-06

**Authors:** Hans Olsson, Per Hultman, Nastaran Monsef, Johan Rosell, Staffan Jahnson

**Affiliations:** ^1^Molecular and Immunological Pathology, Department of Clinical and Experimental Medicine, Faculty of Health Sciences, Linköping University, Department of Clinical Pathology and Clinical Genetics, County Council of Östergötland, 581 85 Linköping, Sweden; ^2^Regional Cancer Centre, County Council of Östergötland, Linköping, Sweden; ^3^Division of Urology, Department of Clinical and Experimental Medicine, Faculty of Health Sciences, Linköping University, Department of Urology, County Council of Östergötland, Linköping, Sweden

## Abstract

*Background and Objective*. The cell cycle is regulated by proteins at different checkpoints, and dysregulation of this cycle plays a role in carcinogenesis. Matrix metalloproteinases (MMPs) are enzymes that degrade collagen and promote tumour infiltration. The aim of this study was to evaluate the expression of various cell cycle regulators and MMPs and to correlate such expression with progression and recurrence in patients with stage T1 urothelial carcinoma of the bladder (UCB). *Patients and Methods.* This population-based cohort study comprised 201 well-characterized patients with primary stage T1 urothelial carcinoma of the bladder. Immunohistochemistry was performed on formalin-fixed material to quantify expression of cell cycle regulators and two MMPs. *Results*. Normal expression of p53 and abnormal expression of MMP9 were associated with greater risk of tumour recurrence. Also, normal p16 expression was related to a lower risk of tumour progression. MMP2, p21, cyclin D1, and pRb showed no significant results that could estimate progression or recurrence. *Conclusions*. Normal p16 expression is associated with a lower risk of tumour progression, but immunohistochemistry on cell cycle regulators and MMPs has little value in predicting the prognosis in stage T1 UCB.

## 1. Introduction

Prognostic factors in patients with superficial (stage Ta and T1) urothelial carcinoma of the bladder (UCB) have been the subject of several publications [1–6]. Depending on the patient and tumour characteristics, the probability of recurrence within one year after transurethral resection (TUR) ranges from approximately 15% to 70% [2], and the likelihood of progression within five years varies from about 7% to 40% [6].

UCB is a heterogeneous disease, and this is particularly apparent in stage T1. Clinical parameters and histopathological findings have only a limited capacity to predict the prognosis, although many studies have demonstrated that such prediction can be achieved by determining the presence of lymphovascular invasion (LVI), tumour grade, and T1 substage [7, 8].

The cell cycle is largely controlled by cell cycle regulators (proteins) at the Gap 1 S-phase and Gap 2 mitosis checkpoints. Dysregulation of the cell cycle at those mentioned steps may trigger carcinogenesis. Immunohistochemical analysis of different cell cycle regulators has helped to explain the molecular pathogenesis of UCB, and, to some extent, it has also had a prognostic impact [9–13]. Many interesting cell cycle regulators can be evaluated by immunohistochemistry (IHC) performed on paraffin-embedded tumour material [9–11].

The current study included a well-characterized cohort of patients who presented with primary stage T1 UCB and were followed for at least ten years or until death. Previous reports from our group indicate that LVI was associated with progression while this was not the case for clinical and other histopathological variables or HER2 immunohistochemical staining [14, 15]. We have now investigated a panel of biomarkers, visualization was achieved by IHC on whole sections of tumour material opposed to tissue microarrays (TMAs), the latter having been shown to provide inferior concordance [16]. We paid special attention to well-known cell cycle regulators, such as cyclin D1, p53, pRb, p21, and p16. The protein p16 is a cyclin-dependent kinase (CDK) inhibitor that controls the rate of the cell cycle via inactivation of the CDK that phosphorylates Rb. The molecules p53 and p21 are tumour suppressors that are involved in carcinogenesis, and cyclin D1 aids cellular processes during the S phase [17]. Matrix metalloproteinases (MMPs) are enzymes involved in the breakdown of extracellular matrix in normal physiological processes, as well as in diseases. It is assumed that MMPs promote tumour infiltration by degrading type IV collagen, the major structural component of basement membranes [18, 19]. 

The aim of the present study was to evaluate the expression of MMPs and different cell cycle regulators, which play important roles in carcinogenesis and tumour progression. This was done to estimate the association of these proteins with the risk of recurrence and progression in a well-characterized population-based cohort of patients with primary stage T1 UCB.

## 2. Patients and Methods

### 2.1. Study Population

We conducted a population-based cohort study. Initially, 285 patients were identified in the Bladder Cancer Registry of the Southeast Healthcare Region of Sweden and were enrolled in the investigation. All the patients were registered as having had a first-time diagnosis of primary stage T1 UCB of transitional cell type between 1992 and 2001 (inclusive). After reevaluation, 201 patients remained in the study population. The reasons for noninclusion were as follows: 52 had a change in T-stage (mainly to Ta) and 32 had either missing specimens or no followup. 

### 2.2. Hospital Records

The patients' hospital records were retrospectively reviewed very carefully with regard to tumour size (two groups: ≤30 mm and >30 mm), multiplicity, and any histologically proven recurrence and progression. Death due to UCB was also recorded. Progression was defined as recurrence with infiltration to T2 or further, regional lymph node involvement, distant metastasis, or death from bladder cancer. Treatment modalities were also found in the hospital records. None of these patients had had intravesical treatment before the first recurrence. A second resection was not done routinely but was performed more often during the latter part of the study period. Patients who developed non-muscle-invasive recurrence in the bladder (*n* = 39) were given one course of induction intravesical BCG treatment for 6 weeks, and, later in the study period, maintenance BCG treatment was also used in some cases (*n* = 12). Progression to a muscle-invasive tumour in the bladder was generally treated by cystectomy or radiotherapy with curative intent. The goal was to have at least 10 years of followup time. 

### 2.3. Histopathological Reevaluation

Tumour specimens were microscopically reevaluated by a uropathologist (HO). The original slides were examined regarding T-stage (presence of deep muscle in the specimens was required for inclusion in the study). As described above, after the initial exclusions, the study population comprised 201 stage T1 patients, and these were subject for further classification concerning WHO grade and eventual presence of LVI. LVI was assessed on the routine hematoxylin-eosin-stained sections, and three different groups were discerned: LVI present, suspected LVI, and LVI not present. LVI was defined as tumour cells within or attached to the wall of a vascular space [7]. It was necessary to include the group with suspected LVI, because retraction artefacts were observed on some of the slides.

### 2.4. Immunohistochemistry

IHC was performed on 4-*μ*m whole sections obtained from each patient's tissue blocks, which had originally been routinely processed by formalin fixation and embedding in paraffin. The blocks were chosen carefully, paying attention to tumour volume and the quality of the embedded material. The tissue sections were deparaffinized in xylene and then rehydrated, pretreated with Tris-EDTA buffer (pH 9) or citrate (only for pRb), and thereafter stained in an automated immunostainer (DAKO TechMate-TM Horizon, DAKO Denmark A/S). A monoclonal mouse antibody was used for all the antigens investigated (see Table 1). Appropriate positive and negative controls were employed throughout. All antibodies were initially individually optimized with respect to the best pretreatment method and dilution.

**Figure 1:**
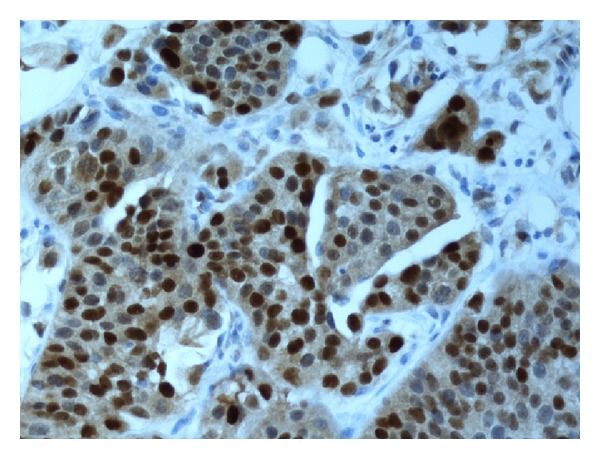
Expression of cyclin D1 (51–75%) in tumour cells.

**Figure 2:**
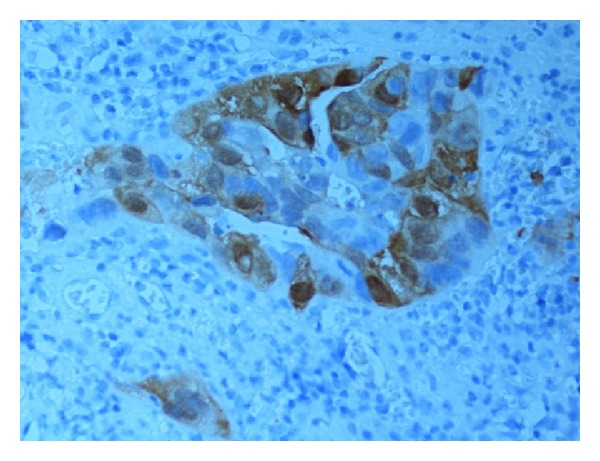
Expression of p16 (51–75%) in tumour cells.

**Figure 3:**
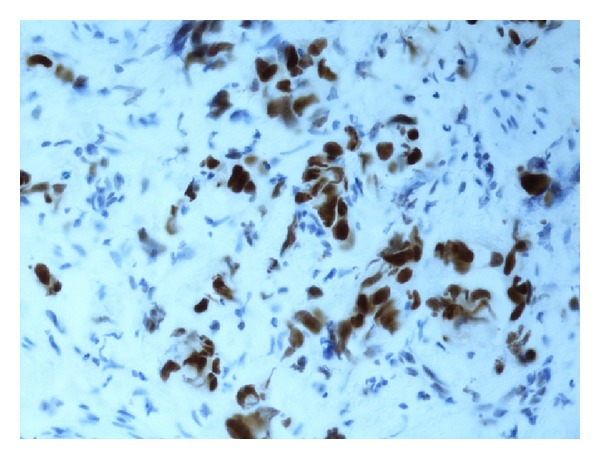
Expression of p53 (100%) in tumour cells.

**Figure 4:**
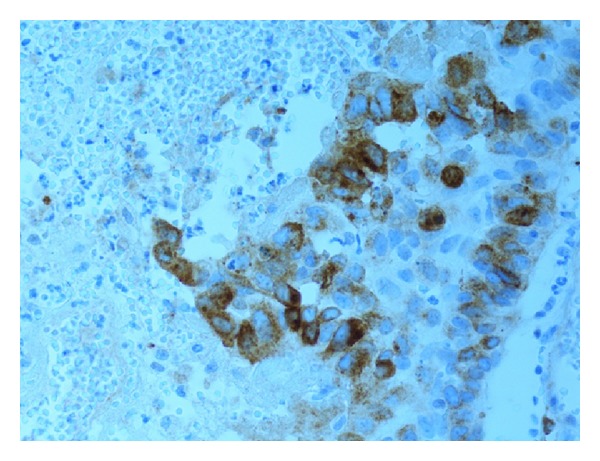
Expression of MMP2 (51–75%) in tumour cells.

Evaluation of the immune staining was done by one pathologist (H. Olsson). As a quality control, one quarter of the study material (i.e., 50 tumours) was investigated independently by another pathologist (N. Monsef). Expression levels of all the antibodies were determined semiquantitatively based on the fraction of tumour cells showing positive staining (0%, 1–10%, 11–25%, 26–50%, 51–75%, 76–100%). Only nuclear staining was used for pRb, cyclin D1 (see Figure 1), and p21; both nuclear and cytoplasmic staining were taken into account for p16 (see Figure 2) and p53 (see Figure 3); only cytoplasmic staining was considered for MMP2 (see Figure 4) and MMP9. For further statistical analysis, all markers were assigned to one of two categories: normal (wild type) or abnormal (altered). The cut-off values were chosen from the studies in the literature and are summarized in Table 1 [11, 12, 18, 20–24].

### 2.5. Statistical Analyses

Comparisons between groups were done using the chi-square test. Cox proportional hazards analysis performed in a univariate and a multivariate fashion was used to analyze different independent variables in relation to recurrence, progression, and death from bladder cancer. It was assumed that there is substantial biological correlation between p21, pRb, and p53, and thus combinations of these three antibodies were also subjected to statistical evaluations. All statistical analyses were performed using IBM/SPSS version 19.0. *P* values of ≤0.05 were assumed to be statistically significant, and all tests were two sided.

## 3. Results

### 3.1. Study Population

The 201 patients in the study population had a median age of 73 years (range 42–93 years) at the time of diagnosis, and 34 (17%) were female. In all, 161 (80%) suffered recurrences, and 77 (38%) had tumour progression. It was our intention to follow the patients for at least 10 years, but the actual follow-up time ranged from 4 to 192 months (median of 60 months). Periods shorter than 10 years were due mainly to high age, other serious diseases, or death from UCB or some other cause. The characteristics of the patients and their tumours are summarized in Table 2.

### 3.2. Immunohistochemistry

All the tumour material from the 201 patients could be evaluated by IHC analysis, and we noted generally good staining results and no doubtful cases. The MMPs tested were usually clearly abnormal (see Figure 1) or clearly normal. MMP2 and MMP9 were abnormal in 18 (9%) and 38 (19%) of the tumours, respectively. Expression of p53 was abnormal in as many as 152 (76%) of the tumours; for this protein, we considered both nuclear and cytoplasmic staining and observed that none of the cases were positive only in the cytoplasm, and, on the whole, very few were positive in the cytoplasm. PRb was abnormal in 168 (86%), p16 in 98 (49%), p21 in 151 (75%), and cyclin D1 in 143 (71%) of the tumours. Table 2 summarizes the results of the IHC analysis and also describes outcome in relation to progression and recurrence.

The quality control of one-quarter of the material (i.e., 50 tumours) by two independent uropathologists resulted in 100% agreement (Kappa 1.0) concerning the breakpoints for abnormal and normal expression of the proteins. There were minor discrepancies between the two pathologists for some samples, but not regarding the intervals for normal and abnormal outcome that had been set before beginning the analysis. 

### 3.3. Statistical Analyses

The results of the statistical analyses are given in Tables 3 and 4. Normal expression of p53 was significantly associated with a higher risk of tumour recurrence, and normal p16 expression was related to a lower risk of tumour progression. Considering the MMPs, abnormal expression of MMP9 was significantly associated with a higher risk of recurrence. In addition to the results of the IHC staining, the multivariate analysis gave results that were statistically significant for tumour size >3 cm and the presence of vascular invasion in relation to recurrence, and vascular invasion was also significantly associated with tumour progression [14].

The statistical analyses of combinations of factors (pRb, p16, p53, and p21) revealed no significant relationship (data not shown).

## 4. Discussion 

We investigated a population-based cohort of primary T1 UCB patients with an essentially natural course of the disease, while none of the patients had received intravesical treatment before the first recurrence (such therapy was not routine in the care region at the time the cohort was established). Using a long follow-up time as in this study is particularly favourable when investigating UCB, which is a long-lasting disease that often involves late recurrences and progression. Previous results have been published by our group concerning standard clinical and pathological features as well as HER2 immunohistochemical staining [14, 15].

Despite the emergence of new diagnostic tools, for instance, in molecular pathology, stage T1 UCB is still a highly unpredictable disease, and it is difficult to make prognoses for individual patients. It is plausible that applying IHC to cautiously selected proteins will identify prognostic factors. Many researchers [10, 12, 13, 21] have described the possibility of performing IHC to analyze cell cycle regulators such as p53, p16, p21, pRb, and cyclin D1, indicating that the levels of expression of these proteins, separately or in combinations, can be exploited as prognostic factors. In a review article, Bolenz and Lotan [25] stated that, at present, no single marker can predict the outcome of UCB and biomarkers derived from the pathogenesis of UCB can be considered to find patients at risk for disease progression.

Our results concerning the proteins we studied as prognostic variables are not encouraging. The multivariate analysis revealed almost no associations between the tested proteins and prognosis, although there were a few exceptions. Expression of p53 was abnormal in as many as 152 cases (76%), and normal expression of this protein was related with a higher risk of recurrence. It is possible that these results were influenced by the paucity of tumours with normal p53 levels. In contrast to our observations, other authors have observed a relationship between abnormal p53 expression and worse prognosis and a higher recurrence rate, as well as a shorter time to recurrence [13, 20]. On the other hand, Peyromaure et al. [26] reported that their results concerning T1G3 bladder cancer and p53 were not statistically significant. The protein p53 has been investigated extensively, and it is a matter of controversy whether IHC analysis of p53 alone can estimate possible abnormality of this molecule. Many studies have shown poor correlation between p53 gene mutations and IHC results [27, 28]. Nonetheless, to some extent, performing IHC to measure p53 expression is considered to be useful for estimating the aggressiveness of many other types of tumours, as has been summarized very well in a review published by Matsushita et al. [9]. On the other hand, at least theoretically, it can be more appropriate to measure levels of a protein by IHC than to analyze defects in its gene. In the present study, we chose to investigate both nuclear and cytoplasmic positivity for p53 and found that none of the cases were positive solely in the cytoplasm, and only a very small number of the tumours showed any cytoplasmic positivity at all. Other authors have often described a lower frequency of p53-positive IHC in UCB than the rate seen in our study. However, the cutoff used by some of those researchers was 20% as compared to 10% in our investigation [29], which might partly explain the high frequency of p53-positive tumours in our cohort. On the other hand, many of the tumours we studied were clearly positive, and only a small number showed 10–25% positivity, although a higher cut-off value might have given another result.

The p16 gene is frequently mutated in cancer, in many cases just as often as seen in the more well-known gene encoding the tumour suppressor p53 [30]. The main function of p16 is to serve as a negative regulator of the cell cycle by binding to and inhibiting cyclin-dependent kinase 4. Accordingly, a nonfunctioning p16 protein disturbs this regulatory effect and thereby favours uncontrolled cell proliferation. Krüger et al. [31] used TMAs to evaluate p16 and p53 (and Ki67) in 73 cases of stage T1 UCB and their results showed an association between tumour progression and abnormal p16 expression in patients with minimally invasive UCB. In our study, normal p16 expression was related to a lower risk of tumour progression. Thus the findings reported by Krüger and colleagues and our results indicate equivalent outcomes, even though different cut-off values were used in the two studies: we set 0% or >50% p16 as abnormal, and Krüger et al. regarded <10% as abnormal. Shariat et al. [23] used the same cut-off level for p16 as we did, and they demonstrated that a combination of p16 and pRb was a marker of, among other things, association with muscle-invasive disease. However, Lee et al. [20] also used the same cut-off value as we did, but they did not detect any statistically significant relationships between p16 and prognosis. Moreover, Benedict et al. [22] have reported a correlation between pRb and p16 expression in UCB, which further supports the use of the analogous cut-off levels for pRb and p16 that we applied.

Cyclin D1 was abnormal in 71% of the tumours in our study, which is comparable to the results reported by Tut et al. [29] showing 83% abnormal tumours even though different cut-off levels were used in the two investigations. Tut and coworkers observed a correlation between cyclin D1 expression and WHO tumour grade (i.e., cyclin D1 positivity was detected more often in grade 3 than grade 1 lesions), but they did not find any significant association between cyclin D1 expression and tumour recurrence and progression.

As mentioned above, pRb is often used in combination with p16. We analyzed various combinations of markers, including p16 and pRb but did not observe any significant results between prognosis and these two proteins combined or other combinations we tested. In contrast, Shariat et al. [23] have shown that p16 together with pRb can serve as a useful marker.

Shariat et al. [23] also noted that 49% of the tumours in their study exhibited abnormal p21 expression. By comparison, we found that 76% of the tumours in our cohort showed abnormal p21 levels. Shariat and colleagues investigated tumours from cystectomy, some of which were stages Ta and T1, but the majority were stage T2 or higher. Despite an assumed difference between more aggressive muscle-invasive tumours and superficial tumours, these authors did not observe any differences in the rate of p21 expression between the two groups (Ta/T1 and T2) of tumours. Shariat et al. also tested p21 in combination with p53 and found some significant associations with prognosis in a selected group of patients.

We also examined the expression of MMP2 and MMP9, which are known to play a role in tumour invasiveness, LVI and induce angiogenesis in several types of cancer [18, 24]. We did find that abnormal expression of MMP9 was associated with a higher risk of recurrence, although, in general, MMP2 and MMP9 showed only weak association with prognosis in the cohort we investigated. 

We have used whole-section IHC, not TMA, the latter of which has been shown to be unpredictable in other studies. Recently, Gudjonsson et al. [16] described using IHC to evaluate the protein epidermal growth factor receptor (EGFR) in both TMAs and whole sections, and the results differed between the two approaches. Accordingly, these investigators questioned whether the assessments of protein expression in TMAs can be generalized. This uncertainty is also indicated by another study in which tumour mapping showed that immunostaining was heterogeneous and that many slides of p53- and p21-abnormal tumours displayed regions with normal immunostaining [32]. 

IHC is an appealing technique, because it allows direct visualization of the results. Furthermore, this approach makes it possible to ensure that it is actually tumour material that is being investigated. IHC is also fairly easy to perform in a routine histopathological laboratory, and it is inexpensive compared to more sophisticated techniques.

## 5. Conclusions

The cell cycle is intricately organized and is controlled by protein complexes. Cancer-related alterations of the expression and functions of specific proteins constitute an integrated result of multiple processes that play important roles in tumour progression and recurrence. Despite massive research efforts in this field over more than three decades, much remains to be investigated, and, thus far, IHC analysis of cell cycle regulators and MMPs has been of little value for estimation of prognosis in stage T1 UCB.

## Figures and Tables

**Table 1:** Antibodies for immunohistochemistry.

Antibodies	Clones	Source	Dilution	Abnormal	Positive
Cyclin D1	P2D11F11	Novocastra	1 : 10	>10%	Nuclei
p53	DO-7	DAKO	1 : 100	>10%	Nuclei and cytoplasm
p21	SX118	DAKO	1 : 50	<10%	Nuclei
p16	6H12	Novocastra	1 : 20	0% or >50%	Nuclei and cytoplasm
pRb	G3-245	BD Pharmingen	1 : 100	0% or >50%	Nuclei
MMP 2	17B11	Novocastra	1 : 40	>10%	Cytoplasm
MMP 9	2c3	Novocastra	1 : 40	>10%	Cytoplasm

**Table 2:** Characteristics of the 201 patients included in the study.

	Recurrence T1		Progression	
	No 40 (20%)	Yes 161 (80%)	No 124 (62%)	Yes 77 (38%)
Gender				
Male	34 (21%)	132 (79%)	102 (62%)	64 (38%)
Female	6 (17%)	29 (83%)	22 (61%)	13 (39%)
WHO 99				
Grade 1	0	0	0	0
Grade 2	4 (12%)	26 (78%)	20 (67%)	10 (33%)
Grade 3	36 (21%)	135 (79%)	104 (61%)	67 (39%)
Tumour size				
≤30 mm	24 (26%)	71 (74%)	60 (63%)	35 (37%)
>30 mm	16 (15%)	90 (85%)	64 (60%)	42 (40%)
Multiplicity				
Yes	32 (23%)	110 (67%)	86 (61%)	56 (39%)
No	7 (12%)	51 (88%)	38 (64%)	21 (36%)
p53				
Normal	4 (8%)	45 (92%)	26 (53%)	23 (47%)
Abnormal	36 (24%)	116 (76%)	98 (64%)	54 (36%)
p16				
Normal	24 (23%)	79 (77%)	70 (68%)	33 (72%)
Abnormal	16 (16%)	82 (84%)	54 (55%)	44 (45%)
pRb				
Normal	6 (18%)	27 (82%)	14 (42%)	19 (58%)
Abnormal	34 (20%)	134 (80%)	110 (65%)	58 (35%)
Cyclin D1				
Normal	15 (24%)	47 (76%)	32 (53%)	30 (47%)
Abnormal	29 (20%)	114 (80%)	96 (67%)	47 (33%)
p21				
Normal	7 (12%)	42 (88%)	32 (54%)	17 (46%)
Abnormal	32 (21%)	119 (79%)	92 (61%)	59 (39%)
MMP2				
Normal	38 (21%)	147 (79%)	114 (63%)	71 (37%)
Abnormal	4 (22%)	14 (78%)	12 (67%)	6 (33%)
MMP9				
Normal	35 (21%)	128 (79%)	104 (64%)	59 (36%)
Abnormal	5 (13%)	33 (87%)	20 (53%)	18 (47%)

**Table 3:** Univariate and multivariate Cox proportional hazards analysis of recurrence after primary transurethral resection for T1 bladder carcinoma in the southeast healthcare region in Sweden 1992–2001.

	Univariate hazard ratio (95% CI)	Multivariate hazard ratio (95% CI)	*P* value**
Tumor size			
≤30 mm	1.0	1.0	
>30 mm	1.48 (1.09–2.03)	1.51 (1.09–2.09)	**0.013**
LVI*			
No	1.0	1.0	
Suspected	1.41 (0.98–2.04)	1.31 (0.88–1.96)	0.19
Yes	2.63 (1.48–4.66)	2.65 (1.43–4.93)	**0.002**
p16			
Abnormal	1.0	1.0	
Normal	0.84 (0.61–1.15)	0.92 (0.66–1.27)	0.61
Cyclin D1			
Abnormal	1.0	1.0	
Normal	0.91 (0.64–1.28)	0.95 (0.67–1.37)	0.80
p21			
Abnormal	1.0	1.0	
Normal	1.07 (0.75–1.53)	1.18 (0.80–1.77)	0.40
p53			
Abnormal	1.0	1.0	
Normal	1.71 (1.21–2.42)	1.58 (1.09–2.28)	**0.015**
MMP2			
Abnormal	1.0	1.0	
Normal	0.80 (0.55–1.16)	0.79 (0.52–1.20)	0.27
MMP9			
Abnormal	1.0	1.0	
Normal	0.69 (0.46–1.03)	0.64 (0.42–0.99)	**0.046**
pRb			
Abnormal	1.0	1.0	
Normal	0.92 (0.60–1.41)	1.00 (0.64–1.56)	1.0

*LVI: lymphovascular invasion.

**P values for variables are from multivariate Cox regression after adjusting for each other.

**Table 4:** Univariate and multivariate Cox proportional hazards analysis of progression after primary transurethral resection for T1 bladder carcinoma in the southeast healthcare region in Sweden 1992–2001.

	Univariate hazard ratio (95% CI)	Multivariate hazard ratio (95% CI)	*P* value**
Tumor size			
≤30 mm	1.0	1.0	
>30 mm	1.28 (0.82–2.01)	1.32 (0.83–2.09)	0.24
LVI*			
No	1.0	1.0	
Suspected	0.99 (0.57–1.73)	0.91 (0.51–1.64)	0.75
Yes	2.75 (1.40–5.41)	3.41 (1.61–7.24)	**0.001**
p16			
Abnormal	1.0	1.0	
Normal	0.51 (0.31–0.83)	0.46 (0.27–0.76)	**0.003**
Cyclin D1			
Abnormal	1.0	1.0	
Normal	0.65 (0.41–1.04)	0.82 (0.49–1.35)	0.43
p21			
Abnormal	1.0	1.0	
Normal	1.52 (0.88–2.64)	1.43 (0.80–2.53)	0.23
p53			
Abnormal	1.0	1.0	
Normal	1.29 (0.78–2.13)	1.41 (0.83–2.39)	0.20
MMP2			
Abnormal	1.0	1.0	
Normal	0.90 (0.52–1.54)	0.82 (0.47–1.45)	0.50
MMP9			
Abnormal	1.0	1.0	
Normal	0.79 (0.45–1.39)	0.89 (0.49–1.59)	0.69
pRb			
Abnormal	1.0	1.0	
Normal	1.20 (0.66–2.18)	1.29 (0.70–2.38)	0.42

*LVI: lsymphovascular invasion.

**P values for variables are from multivariate Cox regression after adjusting for each other.
